# Successful Management of Spontaneous Quadruplet Pregnancy: A Case Report 

**Published:** 2018-09

**Authors:** Leili Hafizi, Elham Rezaii Asgarieh, Nayereh Taheri, Nayereh Ghomian

**Affiliations:** Department of Obstetrics and Gynecology, Imam Reza Hospital, Faculty of Medicine, Mashhad University of Medical Sciences, Mashhad, Iran

**Keywords:** Multiple Pregnancy, Quadruplet Pregnancy, Multiple Gestations, Quadriamniotic Quadrichorionic Placenta, Quadruplets

## Abstract

**Objective:** Triplet or higher-order multiple pregnancies are often caused due to ovulation induction. Spontaneous quadruplet pregnancy is a rare phenomenon which is associated with maternal and fetal complications. Here in, we report a spontaneous quadruplet pregnancy with no family history and as a result of an unwanted pregnancy.

**Case report:** The patient was a 34-year-old, G4 L2 Ab1. She noticed being pregnant during breastfeeding, a spontaneous quadruplet pregnancy. There was no case of multiple pregnancies in her or her husband's family. In week 29 she was hospitalized due to the diagnosis of preterm labour. At 32 weeks and 4 days of gestation, because of the restart of labour contractions and dilatation development, she underwent a cesarean section. The outcome was the birth of 4 healthy neonates weighing between 1800 to 2100 gram and normal Apgar score.

**Conclusion:** Quadruplet pregnancy can rarely occur spontaneously even unintentionally, and can reach the third trimester without prophylactic cerclage.

## Introduction

Since the application of assisted reproduction drugs and technology and the upward shift in maternal age, the prevalence of multiple pregnancies has increased remarkably (1, 2). As maternal and fetal morbidity and mortality are more common in multiple gestations, the successful management of such pregnancies is of great importance (3, 4).

Spontaneous high-order multiple gestation is rare with a prevalence of 0.01 to 0.07% (5, 6). Moreover, the occurrence of spontaneous quadruplet pregnancies is very rare (1, 2, 6); it has a prevalence of 1 in 512,000 to 1 in 677,000 births (7). Up to the year 1999 only 128 quadruplet pregnancies were reported worldwide (8).

Herein, we report a case of a quadruplet pregnancy in which no family history of multiple gestations and no use of assisted reproduction drugs or assisted reproductive technology (ART) was present. It was the outcome of an undesired pregnancy which terminated in the third trimester with 4 healthy neonates and no need for prophylactic cerclage.

## Case report

The patient was a 34-year-old woman, G4 L2 Ab1 who had married her cousin 7 years ago. She had a history of two normal vaginal deliveries and one abortion in the 1^st^ trimester. The first pregnancy in the age of 28 had terminated with a normal vaginal delivery (NVD) resulting in a term baby girl who weighed 3150 gr. The second pregnancy had occurred two years later; curettage was done at week 6 due to spontaneous abortion. Her 3^rd^ pregnancy was in the age 32 resulting in a healthy term baby girl weighing 3400gr with NVD. Due to her unwillingness for becoming pregnant she had withdrawal contraception, whereas because of the non-occurrence of menstruation during breastfeeding and 6 months after her last pregnancy, a pregnancy test was requested. Due to the positive pregnancy result, ultrasound study was done which revealed a 10-week spontaneous gestation with 4 gestational sacs and 4 fetuses. There was no case of multiple pregnancies in her or her husband's family.

She received prenatal care during her pregnancy but there was no need for prophylactic cerclage. At week 24 of gestation she was hospitalized due to premature contractions. The contractions were controlled with the prescription of pethidine and hydration and she was discharged 3 days later. She was once again admitted at 28 weeks of gestation due to similar contractions; this time she was treated with indomethacin and pethidine and discharged 3 days after the contractions suppression. She also received two doses of betamethasone during hospitalization. 

She was admitted a week later due to labour contractions. In vaginal examination 2 finger dilatation with no effacement was detected. Serum test results were reported all in the normal range and the vital signs during hospitalization were normal. At this stage she was treated with tocolytics (adalat). The fetuses' health was monitored by Doppler ultrasound imaging, biophysical profile and fetal non stress test (NST). After the labour contractions' suppression and due to the presence of sporadic contractions she was monitored while being hospitalized up to the time of delivery.

**Figure 1:**
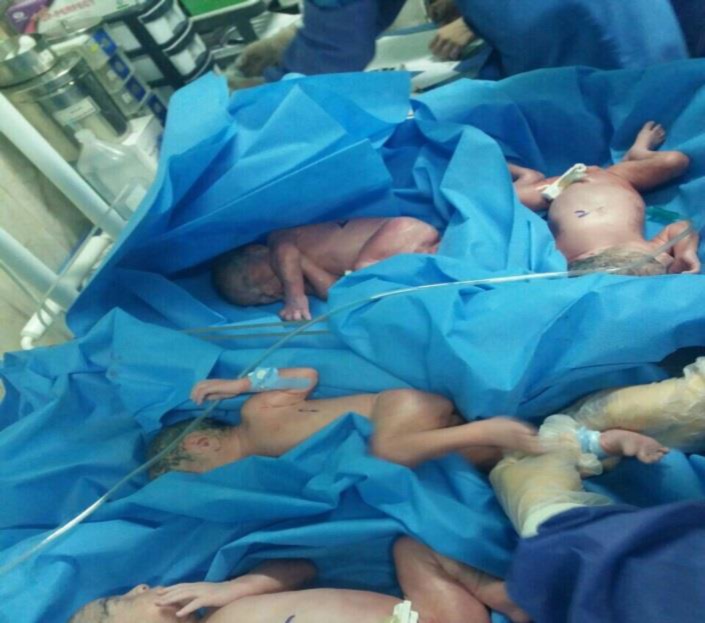
Just born Babies

**Figure 2:**
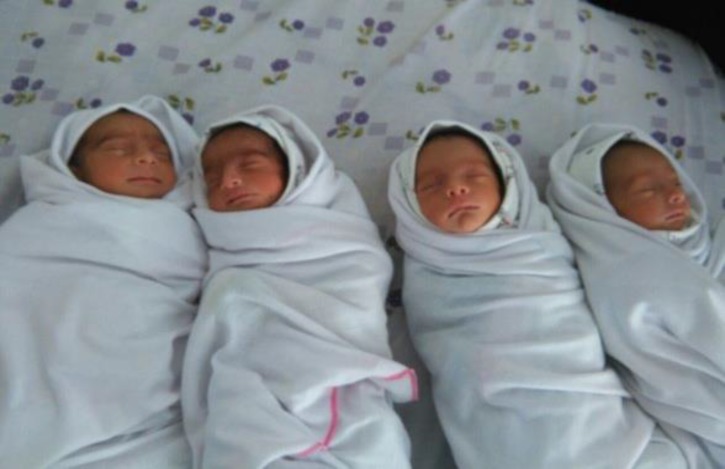
Quadruplet newborn after birth

At 32 weeks and 4 days of gestation, due to the resumption of labour contractions and dilatation progression, after receiving the rescue dose of betamethasone, cesarean section and tubectomy (upon the request of the patient and her husband) was performed. The outcome of cesarean section was 4 fetuses, 3 girls and a boy, quadriamniotic and quadrichorionic. Quadruplet A weighed 1820 gram with an Apgar score of 9 to 10; quadruplet B weighed 1810 gram with an Apgar score of 6-7. Quadruplets C and D weighed 2100 and 1980 gram with an Apgar score of 7-8 and 9-10, respectively. Among the 4 neonates, only quadruplet B was transferred to the NICU; she was discharged after 2 days in good health. Figures 1 and 2 show the quadruplets after birth. 

Because of atonic uterus during the cesarean section, after the administration of the appropriate dosage of oxytocin and methylergonovine and 800µgr of rectal misoprostol, the uterine arteries were blocked and the B-Lynch suture was done. No blood transfusion was required for the mother and her hemoglobin (Hb) level 6 hours after the operation was 9 g/dl; her pre-operational Hb level was 10g/dl. The mother was discharged 3 days after delivery with no complications.

**Figure 3:**
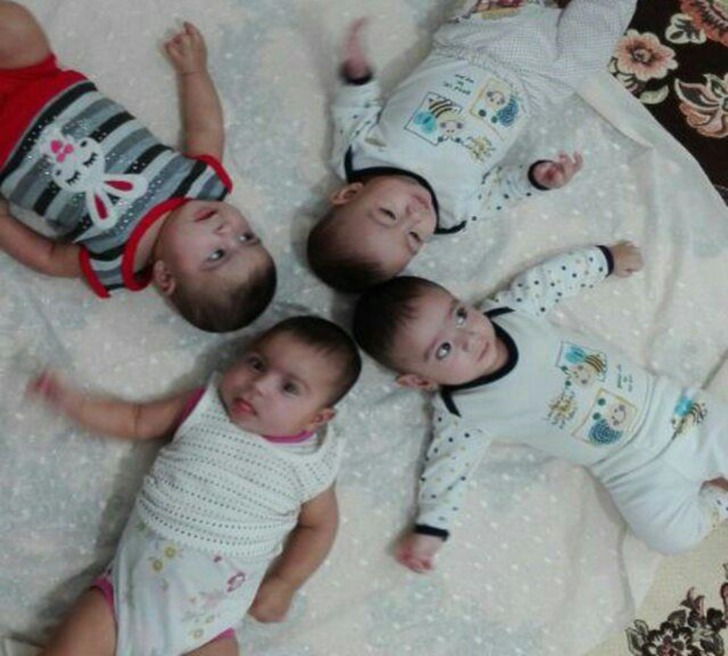
Six months age babies.

For close follow up, the mother and her newborns were visited two weeks after delivery; they were all healthy and had no problem. The infants were visited once again 6 months later revealing normal physical and mental development in all four. Figure 3 shows the babies at 6 months of age.

This project has been approved by Ethical Committee and Vice Chancellor for Research of Mashhad University of Medical Sciences (97/429008).

## Discussion

In the reported patient, pregnancy was the result of a spontaneous conception while the parents mentioned no case of multiple pregnancies in their families. However, most cases of spontaneous quadruplet pregnancies have reported a positive family history (3,9). In 2009, Carrara et al. reported another case of quadruplet pregnancy in which the patient had had a previous twin pregnancy and was herself a twin herself. Preterm labour at week 34 of gestation had resulted in 4 healthy neonates (10). In the case report by Rathod et al. in 2015, one case of spontaneous quadruplet pregnancy was mentioned following spontaneous conception with no family history of multiple pregnancies (11). Nnadi et al. also reported a spontaneous quadruplet pregnancy following 12 years of infertility with no known etiology and no history of multiple pregnancies (6). 

In the reported patient, the quadruplet pregnancy reached the 3^rd^ trimester without prophylactic cervical cerclage. In 1994 El-Tabbakh et al. reported a case of spontaneous quadruplet pregnancy which was terminated at week 33 with 4 healthy neonates (9). Shrestha et al. in 2016 reported another case of spontaneous quadruplet pregnancy, it had terminated at week 33 with 4 healthy neonates (3). Carrara et al. in 2009 reported a case of spontaneous quadruplet pregnancy in which despite prophylactic cervical cerclage at 23 weeks, the contractions had initiated at week 33 resulting in cesarean section at 34 weeks (10). Nevertheless, in some papers even spontaneous quadruplet pregnancies with no signs of premature labour had undergone an elective cesarean section at term (6, 12). 

Goulet et al. in 2001 reported the mean duration of preterm labour delay as 5 days (13). However, in our reported patient despite the quadruplet pregnancy, labour progression was successfully delayed for 8 weeks. Taherian et al. (2004) also reported a case of spontaneous quadruplet pregnancy in which labour was delayed for 23 days (14).

Some cases of spontaneous quadruplet pregnancy have been accompanied by complications in one of the neonates. Rathod et al. in 2015 reported a case of spontaneous quadruplet pregnancy in a 32-year-old woman in which at 35 weeks of gestation one fetus was born as intra uterine fetal death (IUFD) while the other 3 were healthy (11). Quadruplet pregnancy coexistence with a partial molar pregnancy has also been reported; whereas it seems that the reason for the low number of such reported cases is the high rate of related complications and the final failure of such pregnancies (15). Regarding our patient, all four neonates were born with a good Apgar score and normal weight and had desirable mental and physiologic development at their 6-month visit.In the case report by Taherian et al. one fetus died after birth due to asphyxia (14) whereas one fetus experienced IUFD in the 2^nd^ trimester in the case report by Rathod et al (11). In the reported case by Vikranth et al. following the birth of four neonates, one had the signs of intra uterine growth retardation (IUGR) and vesicorectal fistula (12). However, like our patient, the birth of four healthy neonates has been mentioned in some other reports (3, 6, 10).

## Conclusion

Quadruplet pregnancy can rarely occur spontaneously even unintentionally, and can reach the third trimester without prophylactic cerclage.
